# Primary Tuberculosis of Buccal and Labial Mucosa: Literature Review and a Rare Case Report of a Public Health Menace

**DOI:** 10.1155/2023/6543595

**Published:** 2023-10-05

**Authors:** Shyamkumar Sriram, Shamimul Hasan, Shazina Saeed, Syed Ansar Ahmad, Swagatika Panda

**Affiliations:** ^1^Department of Social and Public Health, Ohio University, Athens, Ohio 45701, USA; ^2^Department of Oral Medicine and Radiology, Faculty of Dentistry, Jamia Millia Islamia, New Delhi, India; ^3^Amity Institute of Public Health & Hospital Administration, Amity University, UP, Noida, India; ^4^Department of Oral and Maxillofacial Surgery, Faculty of Dentistry, Jamia Millia Islamia, New Delhi, India; ^5^Department of Oral and Maxillofacial Pathology, Institute of Dental Sciences, Siksha ‘O' Anusandhan University, Bhubaneswar, Odisha, India

## Abstract

Tuberculosis (TB) is a chronic granulomatous infectious disorder, caused by Mycobacterium tuberculosis. Despite the recent advancements in antitubercular therapy (ATT), it remains a global public health concern. TB is a leading infectious cause of global mortality, second only to coronavirus disease 2019 (COVID-19). TB of the oral cavity is an uncommon occurrence and may be classified as a primary and secondary form. The primary tubercular lesions are extremely rare, as the intact oral squamous epithelium resists the entry of tubercle bacilli. The commonest oral TB lesion is solitary ulceration with undermined edges, usually on the tongue, that does not exhibit healing with conservative therapies. Owing to the atypical presentation, the oral TB lesions often go unnoticed during clinical examination; hence, an oral physician should be familiar with the various oral manifestations of TB. A timely diagnosis coupled with interdisciplinary treatment is the key to combat disease dissemination. This manuscript aims to report a rare case of primary tuberculosis of the buccal and labial mucosa in a 43-year-old immunocompetent male patient. Buccal and labial mucosa are the infrequently affected sites for primary oral TB lesions. A detailed literature search carried out on the Google Scholar and PubMed search engines revealed only fifteen case reports and two case series of primary tuberculosis of the buccal mucosa and labial mucosa.

## 1. Introduction

Tuberculosis (TB) accounts for one of the ancient human diseases and is the most common chronic granulomatous disorder, primarily in developing and underdeveloped nations [1].

The early 80s witnessed a diminution in TB cases due to the bacille Calmette-Guérin (BCG) vaccination and upgraded health care services. However, factors including HIV epidemics, multidrug resistance to anti-TB therapy (ATT), TB dissemination in crowded or unsanitary surroundings, immigration from TB-endemic regions, and deteriorated health care systems resulted in its upsurge [2, 3].

TB is a leading infectious cause of global mortality, second only to coronavirus disease 2019 (COVID-19). It resulted in approximately 10.6 million new patients and 1.6 million deaths in 2021 globally, up from 1.5 million in 2020 to 1.4 million in 2019 [4]. These statistics suggest that the COVID-19 pandemic disrupted decades of global progress in decreasing TB mortality, and the total number of TB-related deaths in 2020 has reverted to the same level observed in 2017 [4]. The lack of effective methods to precisely diagnose latent TB infection (LTBI) and the upsurge in extensively drug-resistant TB (XDR-TB) and multidrug-resistant TB (MDR-TB) cases pose a major challenge to the prevention and management of TB [5, 6].

In 2021, TB cases primarily occurred in Southeast Asia (45%), Africa (23%), Western Pacific (18%), Eastern Mediterranean (8.1%), the Americas (2.9%), and Europe (2.2%). The 30 highest disease-burden nations accounted for 87% of the estimated global incident TB cases. Eight of these countries, namely, India (28%), Indonesia (9.2%), China (7.4%), the Philippines (7.0%), Pakistan (5.8%), Nigeria (4.4%), Bangladesh (3.6%), and the Democratic Republic of the Congo (2.9%), constituted for more than two-thirds of the global TB cases [4]. The disease is primarily seen in developing and underdeveloped nations [7], where an increased exposure to TB bacteria (e.g., close contact with TB patients in a crowded, unhygienic environment, or birth in a TB-endemic area), lack of health, and impaired immunity may increase the risk of TB [8]. In India, the total number of incident TB cases (new and relapse) notified during 2021 was 19,33,381, in contrast to that 16,28,161 in 2020. Thus, there was a notable increase of 19% in the number of TB patients' notifications in 2021 [9].

Tuberculosis can be categorized as pulmonary or extrapulmonary, with pulmonary TB being the most common form [2, 3]. Extrapulmonary lesions can occur in lymph nodes, peritoneal cavity, genitourinary, musculoskeletal, nervous, and hepatosplenic systems, either through self-inoculation via infected sputum, or by hematogenous or lymphatic seeding [2, 3, 10]. Extrapulmonary lesions are infrequent, accounting for 10% to 15% of infected people [2, 3, 11].

Oral tuberculous lesions are rare, with a reported incidence of 0.05% to 5% of all TB cases [2, 12–14]. Most cases occur secondary to pulmonary tuberculosis, while primary lesions are extremely rare [2, 14]. Primary oral TB lesions primarily affect the gingiva of children and young adults and are usually accompanied by regional lymphadenopathy. This contrasts with the secondary oral TB lesions, which are more prevalent among middle-aged and elderly individuals and mainly affect the tongue [2, 12, 14–18].

A timely diagnosis and efficient management of TB are imperative and may be achieved with various diagnostic aids, including radiographic imaging, microbiological tests, immunological response tests, histopathology, and molecular investigations [19, 20]. Oral tuberculous lesions may present as the sole presentation of the disease, posing diagnostic dilemmas and misdiagnosis due to their nonspecific presentation [17, 21]. Hence, oral healthcare professionals should always consider TB in the differential diagnosis of chronic, recalcitrant oral ulcerations [21].

TB is a curable disease treated with a World Health Organization (WHO)-recommended directly observed treatment short (DOTS) course. The therapy consists of an initial 2-month phase of first-line combination therapy with isoniazid (INH), rifampicin (RIF), pyrazinamide (PZA), and ethambutol (E) followed by a continuation phase of 4 months with INH and RIF. Dose interruption in DOTS therapy may produce drug resistance and reoccurrence of the disease.

DOTS interferes with the biosynthesis of mycobacterial cell wall proteins encoded by different genes. Overexpression of these genes may produce drug resistance, either due to inappropriate dosage or the use of compromised-quality antitubercular drug therapy. Despite M. tuberculosis control by first-line combination therapy, multidrug resistance of tuberculosis (MDR-TB) occurs in first-line drugs. Second-line therapy in the form of a 6–9-month DOTS course of aminoglycoside antibiotics such as streptomycin and fluoroquinolones (e.g., ciprofloxacin, sparfloxacin, or moxifloxacin) is given in such cases [22–24].

The emergence of multidrug-resistant (MDR) and extensively drug-resistant (XDR) tuberculosis can be attributed to the adverse effects associated with DOTS (hepatotoxicity, hypersensitivity, and gastric intolerance) as well as poor patient compliance due to the lengthy treatment course, inadequate medication adherence, and inappropriate treatment regimen [25, 26].

This article is aimed at reporting a rare case of primary oral TB manifesting as nonhealing ulcers of the buccal and labial mucosa in a 43-year-old immunocompetent male. A definitive diagnosis of primary oral TB was made based on the history and clinical examination, coupled with histopathology, acid-fast staining, and chest X-ray.

## 2. Case Description

**Figure 1:**
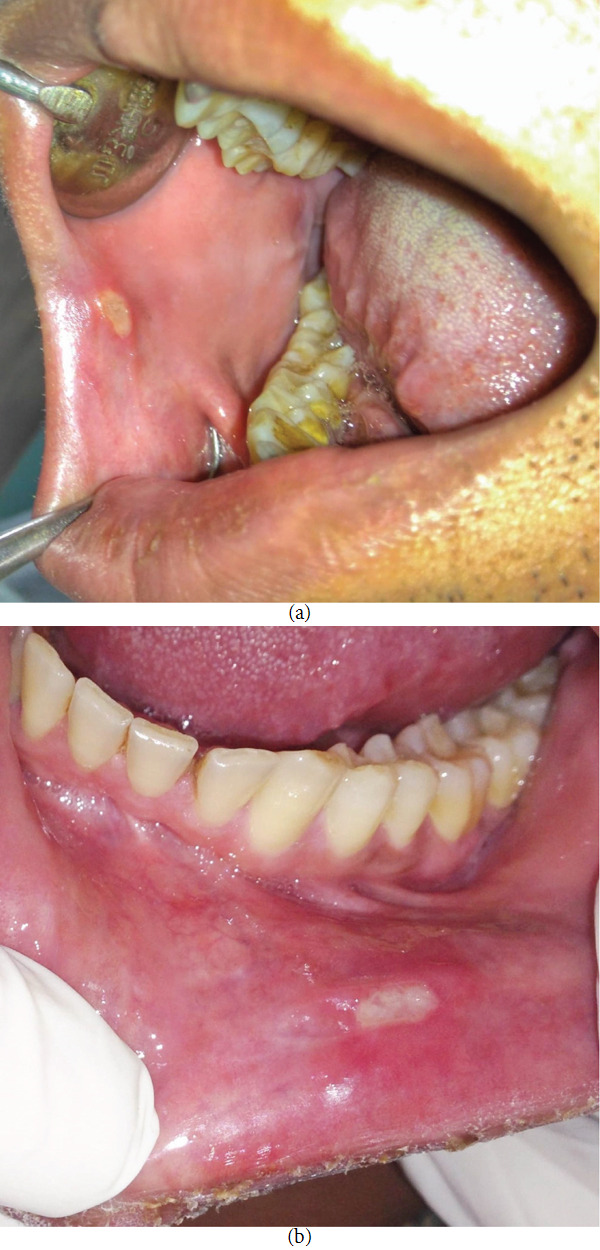
(a) Solitary ulcer on the right buccal mucosa. (b) Ulcer on the left lower labial mucosa.

A 43-year-old immunocompetent male from low socioeconomic status was referred by a public health camp to our outpatient department for the evaluation of persistent, nonhealing ulcers of the buccal and labial mucosa of the lower lip for the last 6 months. History elicited that the patient was asymptomatic 6 months back when he noticed small ulcers (without vesicle formation) in the buccal and labial mucosa of the lower lip, which have gradually reached the present size. The ulcers were initially painless but have become painful over the last 3 months. The medical and family history was nonsignificant, and the patient denied the intake of any systemic medications. Personal history was significant for occasional tobacco chewing, but the patient has quit the habit for more than a year. However, he denied the consumption of alcohol. There was no history of any weight loss, fever, and hemoptysis. The patient consulted a few private practitioners and was prescribed medications. The previous medical prescriptions revealed the use of Metrohex gel (0.25% chlorhexidine gluconate and 1% metronidazole), Orasep gel (choline salicylate and tannic acid), Turbocort oromucosal paste (triamcinolone acetonide, 0.1%), and several antibiotic therapies. However, the ulcers did not respond to conservative therapies. The general physical examination was noncontributory, with no involvement of the lymph nodes. On intraoral examination, a nonhealing ulcer on the labial mucosa of the lower lip on the left side measuring 1.5 cm × 1 cm was seen. Another ulcer, measuring roughly 1 cm × 0.8 cm in diameter, was seen on the right buccal mucosa adjoining the anterior labial commissure, roughly 2 cm from the angle of the mouth and 2 cm below the occlusal plane. The ulcers were oval with distinct, slightly raised borders. The ulcers were covered with a yellowish grayish pseudo membrane and surrounded by mild erythema. The edges of the ulcers were undermined, with the ulcer base being granular and mildly indurated. Mild tenderness on palpation was also elicited (Figures 1(a) and 1(b)).

Considering a chronic nonhealing ulcer, recalcitrant to conservative management, a differential diagnosis including aphthous ulcer, traumatic ulcer, malignant ulcer, drug reaction, and infections (bacterial, fungal, and viral) was made. As the ulcers were persistent, nonrecurrent, with no associated traumatic component, the likelihood of traumatic and aphthous ulcers was ruled out. Ulcers due to drug reactions were ruled out due to a negative history of any systemic medications.

**Figure 2:**
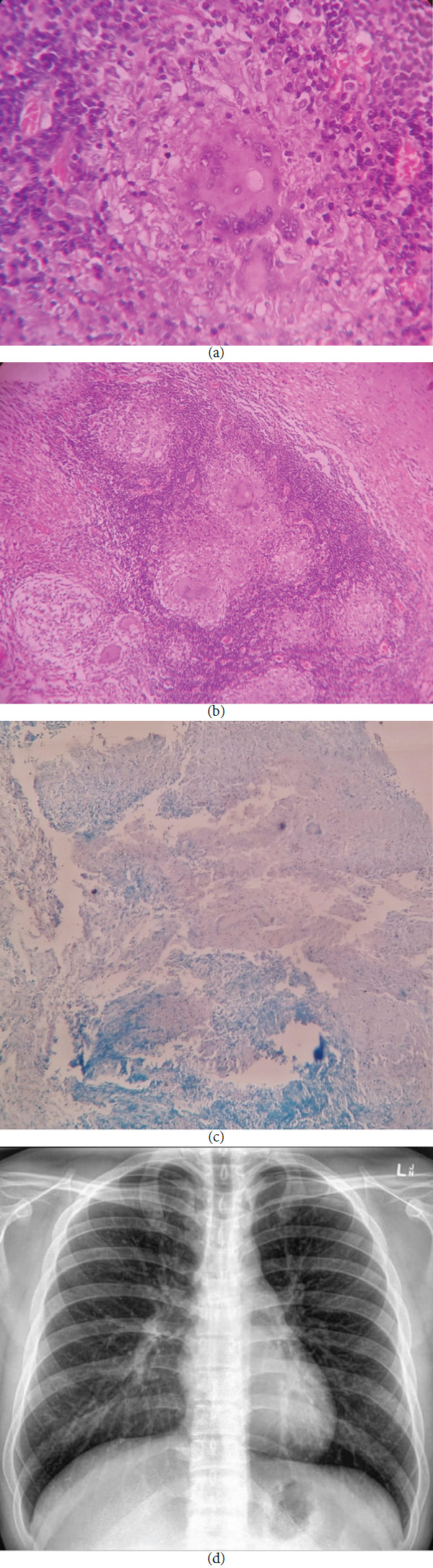
(a) The H and E photomicrograph (10x) shows caseous necrosis, epithelioid cells, and Langhan's giant cells. (b) The H and E photomicrograph (40x) shows chronic inflammatory cells and Langhan' giant cells. (c) The photomicrograph (Ziehl-Neelsen staining; 10x) shows acid-fast mycobacteria suggesting tuberculosis. (d) Chest X-ray revealing a normal picture.

Written consent from the patient was taken, and an incisional biopsy from the ulcer edge was done under local anesthesia. Microscopic examination showed the typical features of a granulomatous lesion (caseating granulomas, encircled by epitheloid cells, Langhan's giant cells, and chronic inflammatory cells) (Figures 2(a) and 2(b)). Ziehl-Neelsen staining (ZN staining) revealed several acid-fast bacilli on a smear examination of the ulcer (Figure 2(c)). Blood investigations were within the normal limits, except for a raised erythrocyte sedimentation rate (35 mm in the first hour of Wintrobe). The hepatitis C virus test, the VDRL (Veneral Disease Research Laboratory), and HIV tests were negative. Chest (PA view) was advised to look for systemic involvement, which revealed a normal picture (no lung infiltrates, lung opacities, bilaterally normal lung fields, and normal bronchovascular markings) (Figure 2(d)). Thus, a confirmatory diagnosis of primary TB of the buccal and labial mucosa was arrived at.

**Figure 3:**
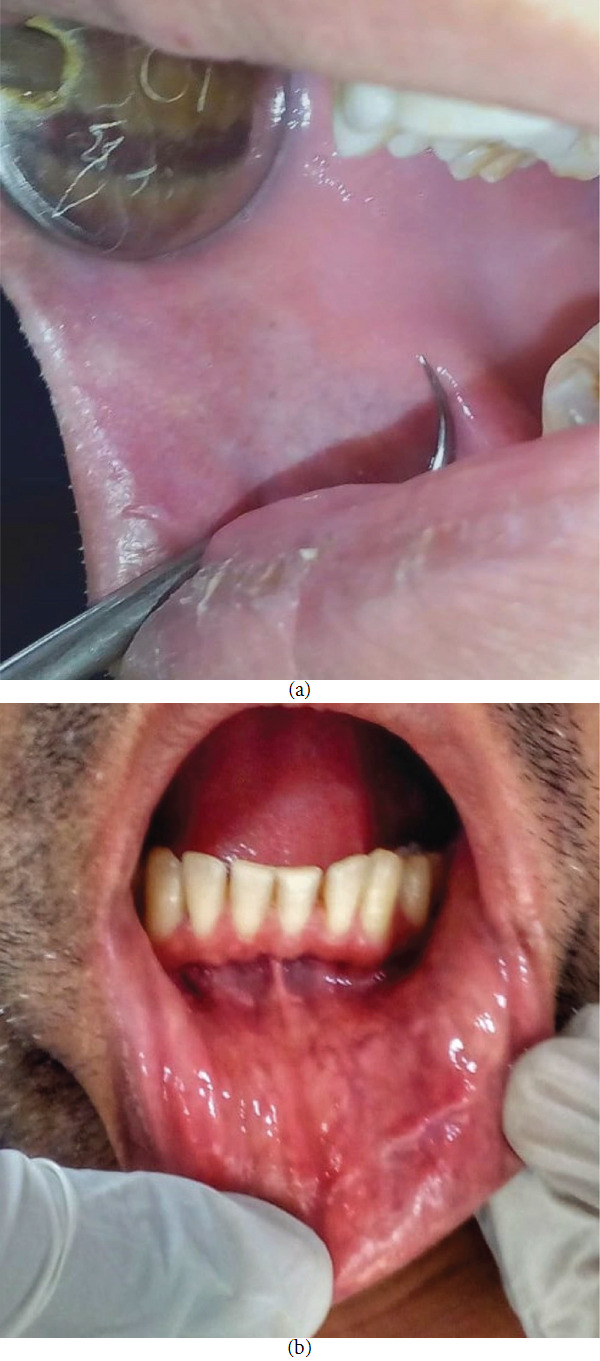
(a) Healed lesion on the right buccal mucosa. (b) Healed lesion on the left lower labial mucosa.

The patient was referred to the Department of Internal Medicine, where he was advised antitubercular treatment (ATT) in 2 phases for a total of 6 months. The oral ulcers showed complete resolution after about 1 month of ATT (Figures 3(a) and 3(b)). No recurrence was reported during the 6-month regular follow-up.

## 3. Discussion

Tuberculosis (TB) refers to a chronic granulomatous infectious disorder caused by Mycobacterium tuberculosis, primarily due to the inhalation of Mycobacterium-impregnated airborne droplets [2, 14, 15, 21, 27–29].

Tuberculosis is the second most common infectious cause of global mortality, surpassing AIDS. According to WHO statistics, globally, approximately 2 billion people are infected with tuberculous bacilli, with an annual 1% increase in TB incidence due to multidrug-resistant M. tuberculosis strains in HIV and AIDS patients [27].

Active TB patients typically display salient constitutional signs and symptoms, such as persistent cough, hemoptysis, fever, weight loss, anorexia, and lymphadenopathy [21, 30]. However, the classic features may be absent in up to 20% of active TB patients (especially geriatric and immunocompromised individuals) [21].

All the above classical features were absent in our patient.

Oral tuberculous lesions are atypical in their clinical presentation and are frequently overlooked [13, 30]. Oral healthcare professionals play a vital role in identifying these unusual oral lesions, thereby diagnosing tuberculosis in individuals who may be unaware of the disease [17].

Oral TB lesions can be primary or secondary, with primary inoculation occurring when tubercle bacilli enter the oral mucosa without prior infection. The role of trauma is debatable, as the oral stratified squamous epithelium remarkably resists direct entry by tubercle bacilli, thus explaining the rarity of oral TB lesions [2, 15, 16, 18, 21, 28, 31]. The exact mechanism of primary inoculation remains obscure, although it has been proposed that chronic inflammation or traumatic episodes may be the most likely factors to breach the integrity of the oral mucosa [15, 28, 32]. In the present case, chronic inflammation due to tobacco chewing may have caused abrasion of the oral mucosa, thus elucidating the most likely portal for primary inoculation of tubercle bacilli.

In the secondary form, oral TB lesions usually occur secondary to pulmonary disease, and bacilli get inoculated in the oral tissues from infected sputum or hematogenous/lymphatic seeding [14, 15, 30, 31]. The differentiating features between primary and secondary tuberculosis are summarized in Table 1.

Over 40% of TB cases typically present with a solitary, indurated, painful ulceration, with ill-defined borders and covered by inflammatory exudates, although unusual cases with multiple lesions or asymptomatic ulcers have also been documented [31]. Oral TB ulcers are chronic, nonhealing, and slowly increase in size [14, 18, 35].

Buccal and labial mucosa are the infrequently affected sites for primary oral TB lesions. A detailed literature search carried out on the PubMed search engine and electronic databases (Scopus, Web of Science) revealed fifteen case reports [17, 21, 35–47], and two case series [34, 48] of primary tuberculosis of the buccal and labial mucosa, as depicted in Table 2.

Differential diagnoses of an oral tubercular ulcer include aphthous ulcers, traumatic ulcers, syphilitic ulcers, and malignant ulcers. TB is given a place in the list of differential diagnoses only when the histologic examination reveals the presence of a granuloma. The other histologic differentials include sarcoidosis, Crohn's disease, deep mycotic lesions, tertiary syphilis, and Melkersson-Rosenthal syndrome [2, 13, 17, 18, 21, 27, 29–31, 36]. A detailed differential diagnosis of oral ulcers is represented in Table 3.

In the present case, ulcers were persistent and nonrecurrent, and there was no associated traumatic component, thus ruling out the likelihood of traumatic and aphthous ulcers. Ulcers due to a drug reaction were excluded based on a negative history of any systemic medications. HIV and sarcoidosis were ruled out by serology and the presence of caseation and AFB on histopathological examination, respectively.

Kakisi et al. reported that a majority of the patients (94%) were unaware of their TB infection. Hence, they recommended a prompt investigation for atypical, chronic, nonhealing oral ulcers [32]. The various investigations employed in the diagnosis of TB are represented in Table 4.

The therapeutic regimen is aimed at eradicating TB and preventing chronic disability, arising from either the disease or as an adverse effect of ATT. Approximately 85% of drug-sensitive TB (DS-TB) have been treated successfully [1]. The recommended 6-month therapy for DS-TB consists of 2 phases: (a) an intensive 2-month regimen of rifampicin (RIF), isoniazid (INH), pyrazinamide (PZA), and ethambutol (ETM), (b) a continuation 4-month therapy of RIF and INH [51].

WHO introduced a “Directly Observed Therapy, Short Course” (DOTS) for global TB control. It primarily focuses on direct patient monitoring by trained staff, thus ensuring patient compliance and minimizing drug resistance risks [18].

Local management of oral TB ulcers includes the elimination of traumatic etiology, the use of anti-inflammatory gels, and maintaining meticulous oral hygiene [14].

Infection-control protocols should be maintained in dental clinics to minimize nosocomial infections and occupational hazards. Proper sterilization protocols, personal protective equipment, and meticulous hand hygiene etiquette should be followed [18]. The use of N95 respirators, rubber dams that minimize aerosol generation, and surface cleaning after every dental procedure should be encouraged [29].

Dental personnel should be educated about the constitutional features of TB. Individuals with active symptoms should be isolated and referred for immediate medical care. Any elective treatment should be deferred until noninfectious, and urgent dental treatment should be carried out in airborne infection isolation facilities. Universal infection control measures should be taken in patients with latent TB [17, 29].

Protein-energy or micronutrient deficiency leads to altered immune-homeostasis, which greatly increases an individual's susceptibility to infections or progression of infection to disease. An array of nutrients like macro- and micronutrients (vitamins, minerals, and trace elements) are associated with boosting the immune responses against intracellular pathogens like M.tb. These nutrients have an immunomodulatory effect in controlling the infection and inflammation process [52].

The inclusion of the “End TB Strategy” (2014) within the Sustainable Development Goals (SDGs) 3 (2015) is aimed at reducing the overall TB incidence and mortality by 90% and 95%, respectively, by 2035, thus strengthening the global fight against TB [51, 53]. The COVID-19 pandemic has immensely hampered the already lagging progress toward reducing the global TB burden. Hence, it is essential to incorporate an integrated plan combining biomedical and social protection interventions for local, regional, and national matters [53].

## 4. Conclusions

Oral primary tubercular lesions are extremely uncommon and pose a diagnostic challenge due to their atypical presentation. Buccal and labial mucosas are rare oral sites of tubercular involvement. A detailed literature search revealed only fifteen case reports and two case series of primary tuberculosis of the buccal and labial mucosa. Our patient reported with oral ulcers recalcitrant to conservative therapies for the last 6 months. Histopathology, acid-fast staining, and chest radiograph established a diagnosis of primary oral tuberculosis. The patient responded well to ATT and showed no recurrences during the 6-month follow-up.

## 5. Recommendations

- Oral primary tuberculous lesions are an uncommon occurrence and often pose a diagnostic threat owing to their nonspecific manifestations
- Tuberculosis should always be given a place in the differential diagnosis of any atypical oral ulcer, recalcitrant to conservative therapies, especially in patients from TB-endemic regions
- An oral physician must be cognizant of the various manifestations of oral TB, thus impeding delayed diagnosis and treatment

## 6. Patient Feedback

The patient experienced uneventful favorable healing after the ATT therapy and was completely satisfied with the treatment protocol. The patient has been carefully examined and followed up for 6 months, during maintenance visits scheduled every 4 weeks. No recurrence of oral ulcers was reported, and the patient was completely asymptomatic during the follow-up period.

## Figures and Tables

**Table 1:** Differentiating features between primary and secondary tuberculosis [2, 14–16, 18, 28–30, 33, 34].

Variable	Primary oral tuberculosis	Secondary oral tuberculosis
Occurrence	Extremely rare, seen primarily in children	More frequently seen than the primary form, primarily in the middle-aged and elderly
Risk factors	(1) Systemic factors (a) Lowered host resistance (i) Primary & secondary immunodeficiency (ii) Nutritional deficiencies (b) Increased virulence of the mycobacteria (2) Local factors: oral mucosal traumas (extraction sockets, jaw fracture), chronic inflammation (poor oral hygiene, tobacco habit, periodontitis, chronic pulpitis, dental abscess/cysts), hyperkeratotic disorders (leukoplakia) (3) General factors: overcrowding, poor ventilation and sunlight source, early marriage, and repeated pregnancies at small intervals	
Clinical Manifestation	Ulcer: superficial or may be larger and deeper	Ulcer: with undermined, irregular edges, covered with Trélat granules
Oral site	Mostly gingiva	Mostly tongue
Pain/soreness	Lesion is painless	Pain
Lymph nodes	Enlarged and tender	Enlarged/not enlarged, and generally nontender
Management	Antitubercular therapy (ATT), topical application of anti-inflammatory gels, and mucosal protecting agents. Maintaining meticulous oral hygiene and removing of plausible traumatic factors	

**Table 2:** Reported cases of primary tuberculosis of buccal and labial mucosa [17, 21, 34–48].

S. no.	Author(s) and year	Age (years)/sex	Oral site affected	Other associated features
1	Sachdeva et al., [17]	12/F	Solitary ulcer (4 × 4 cm) on the left lower labial mucosa	Nonpalpable lymph nodes
2	Verma et al., [21]	34/M	Ulcers on the left inner commissure (3 × 2 cm) and left buccal vestibule (3 × 1 cm)	Chronic smoker and occasional alcohol consumption
3	Ebenezer et al., [35]	40/M	Solitary ulcer on the left buccal mucosa	Enlarged, mobile, nontender left submandibular lymph node; chronic smoker
4	Nanda et al., [36]	35/F	Solitary ulcer (1.5 × 1.5 cm) on the left buccal mucosa	Palpable, mobile, nontender left cervical lymph node
5	Bairagya et al., [37]	46/M	Solitary, discrete ulcer < 1 cm on the left buccal mucosa	—
6	Gokak et al., [38]	21/M	Ulcers on the upper labial mucosa and lip encrustations	Enlarged, erythematous, granular maxillary labial gingiva
7	Saoud ZT et., [39]	16/F	Solitary linear ulcer (3 cm) on the lower labial mucosa	Bilateral submandibular lymphadenopathy and left jugulocarotid adenopathy
8	Hathiram et al., [40]	50/M	3 × 4 × 1 cm ulceroprolifrative growth on the left buccal mucosa	—
9	Besra K et al., [41]	41/M	1.5 × 1 cm ulcer on the upper labial mucosa	5 × 4 cm ulcerative lesion on the hard palate
10	Khammissa RAG et al., [42]	33/F	Painful ulcer on the upper left labial mucosa	HIV-seropositive with a CD4+ T cell count of 429 cells/mm^3^. Ulcer on the tongue
11	Gupta A et al., [43]	24/M	Reddish-pink granular lesion on the vermillion border, labial mucosa, floor of the mouth, and the mandibular anterior gingiva	Diffuse, nontender swelling of the lower lip with mild lip eversion
12	Pulin Saluja et al., [44]	36/F	2.5 × 3 cm ulcer on the left buccal mucosa and vestibule with ill-defined margins	Palpable left submandibular lymph node, firm, and nontender
13	Virad Kumar et al., [45]	4/M	Diffuse, firm upper lip swelling with fissuring on the mucosal surface	Palpable, solitary left submandibular lymph node (2 × 2 cm)
14	Nagabhushan D et al., [46]	10/F	1 × 1.5 cm ulcer on the right retromolar region in the posterior buccal mucosa	Palpable right submandibular lymph nodes, firm, and mobile
15	Awasthi S et al., [47]	40/M	rough, elevated, irregular indurated lesion, 1 × 1.5 cm over the right buccal mucosa	H/O chronic tobacco chewing, malaise and weight loss
16	Rout et al., [34]	6/M	Chronic ulcer (3 × 3 cm) on buccal mucosa	—
17	Wang et al., [48]	36/M	Left buccal mucosa	—
		54/M	Vestibule & left lower lip	—
		27/M	Left lower lip	—

**Table 3:** Differential diagnosis of oral ulcers is presented [14, 28].

Oral disease	No. of ulcers	Pain/soreness	Course & duration	Clinical picture
Oral TB	Single	Primary oral TB—painless, secondary oral TB—painful	Chronic ulcer for **>**3 weeks, chronic cough, haemoptysis	Ragged, indurated, and irregular margins, Trélat granules, cobblestone appearance
Recurrent aphthous stomatitis	Single/multiple	Yes	Recurrent ulcers, spontaneous healing after 7–30 days	Shallow ulcer, inflamed halo
Traumatic ulcers	Single/multiple	Yes	Spontaneous healing after elimination of traumatic factor/institution of anti-inflammatory therapy	Inflamed base, shallow or deep ulcer, margins slightly elevated
Malignant ulcer	Single/multiple	Initially—painless, later—painful	Chronic ulcer, developing slowly	Nodular, punched-out ulcer with irregular margins, indurated base, fixed lymphadenopathy
Syphilis	Single	No	Ulcer lasting for 2–6 weeks, spontaneous healing	Smooth, indurated margins
Histoplasmosis	Single/multiple	Yes	Chronic ulcer for >3 weeks, persistent cough, pulmonary changes	Irregular, indurated margins
Ulcerative lichen planus	Single/multiple	Yes	Recurrent ulcers may be preceded by subepithelial bullas	Shallow ulcer, Wickham's striae present

**Table 4:** Summary of the various employed diagnostic aids in TB [14, 15, 17, 27–29, 31, 34, 36, 49, 50].

Diagnostic tool	Method/inference	Merits	Limitation /drawback
Tuberculin skin Test (TST)/Mantoux test	5 tuberculin units were injected intradermally and read 48-72 hours later. Positive when induration of 5-15 mm is seen	Used as an essential screening diagnostic tool Helpful in the diagnosis of active TB More precise than radiographs Easy to perform	False-positive test results due to cross-reactivity with BCG or non-TB mycobacteria False-negative results in immunocompromised individuals Difficult to use in children Test results are interpreted only after 48-96 hours; thus, a follow-up visit is required

Interferon release assays (IGRAs)	The amount of interferon-gamma (IFN-Y) in response to contact with the TB antigens is measured	Not confounded by previous BCG vaccination Approved by the Food and Drug Administration (FDA) as a more precise substitute to TST for the diagnosis of TB infection	Expensive, poor predictors for TB progression Cannot distinguish between LTBI and active TB

TST or IGRAs alone have a suboptimal ability to rule in or negate active TB. Hence, suitable clinical samples for microbiological and molecular assays should be collected from every patient suspected of active TB. IGRAs should always be employed with other investigations (e.g., TST results and chest X-ray findings) to establish an active TB diagnosis			

Staining (a) Ziehl-Nelson staining (AFB staining) (b) Auramine fluorescence	Acid-fast bacilli (AFB) are seen as bright red rods against a blue, green, or yellow background	Simple method, economical, noninvasive	As there is a relative dearth of tubercle bacilli in oral specimens, the ability to affirm acid-fast bacilli in histological samples is quite low (7.8%) A similar appearance may be seen with saprophytic mycobacteria Requires expensive equipment Used as a screening tool, not for final diagnosis
	Visualizes acid-fast bacilli as bright rods against a dark background using a fluorescent microscope	Contrast bacilli can be readily seen under a high-dry objective More sensitive Less tiring, quick results for a large number of slides	Requires expensive equipment Used as a screening tool, not for final diagnosis

Histopathology Histopathological evaluation is necessary for patients with nonhealing ulcers (of more than 3 weeks) with the absence of constitutional features	Granulomatous disorders may be considered if the histologic examination reveals the presence of granulomas	Gold standard diagnostic aid	Delayed or erroneous histologic diagnosis may be seen as granulomas may not be noticeable in early lesions, or can be absent in immunosuppressed individuals

A combination of acid-fast staining (Ziehl-Neelsen staining) and histopathology can serve as definitive investigative aids for a precise diagnosis			

Radiographs of suspected TB cases should be advised for posterior-anterior (PA) and lateral view chest radiographs, even in the absence of constitutional symptoms	Areas of calcifications, cavities, or radiolucency	Easy to perform Quick interpretation	Exposure to X-rays Poor sensitivity Cannot distinguish between active TB and healed TB in case of scar formation

Culture (a) Lowenstein–Jensen media (LJ media) (b) BACTEC	When grown on LJ media, M. tuberculosis appears as brown granular colonies	Less expensive than BACTEC Fewer chances of contamination	Takes 4-6 weeks to get visual colonies on media No differentiation between M. tuberculosis and other Mycobacterium species
	Detects the presence of oxygen in fluorescence by scanning it after every hour Positive samples may contain 105–10^6^ CFU/ml	Early detection Differentiates M. tuberculosis from other Mycobacterium species More sensitive than conventional LJ media	Expensive More risk of contamination

Polymerized chain reaction (PCR)	Helps in the detection of infectious agents and can differentiate between nonpathogenic and pathogenic strains	Rapid diagnostic aid Easy amplification of even very small-sized DNA High sensitivity, virus detection soon after infection and even before the disease onset	Localization within tissues is not possible Staging of mycobacterial disease is not possible GeneXpert requires professional training and is expensive

## Data Availability

The data used to support the findings of this study are available from the corresponding author upon request.
